# Studies Regarding Antimicrobial Properties of Some Microbial Polyketides Derived from *Monascus* Strains

**DOI:** 10.3390/antibiotics13111092

**Published:** 2024-11-16

**Authors:** Daniela Albisoru, Nicoleta Radu, Lucia Camelia Pirvu, Amalia Stefaniu, Narcisa Băbeanu, Rusandica Stoica, Dragos Paul Mihai

**Affiliations:** 1 Faculty of Biotechnologies, University of Agronomic Sciences and Veterinary Medicine of Bucharest, 59 Marasti Boulevard, District 1, 011464 Bucharest, Romania; 2 Department of Biotechnologies, National Institute of Chemistry and Petrochemistry R&D of Bucharest, 202 Splaiul Independentei Street, District 6, 060021 Bucharest, Romania; 3 Department of Pharmaceutical Biotechnologies, National Institute of Chemical Pharmaceutical Research and Development, Bucharest, 112 Vitan, 031299 Bucharest, Romania; lucia.pirvu1@gmail.com (L.C.P.);; 4 Faculty of Pharmacy, “Carol Davila” University of Medicine and Pharmacy, 6 Traian Vuia Street, 020956 Bucharest, Romania; dragos_mihai@umfcd.ro

**Keywords:** antimicrobial, polyketides compounds, antimicrobial, properties predicted in silico, in vitro validation

## Abstract

Finding new molecules to prevent the growth of antimicrobial resistance is a hot topic for scientists worldwide. It has been reported that some raw bioproducts containing *Monascus* polyketides have antimicrobial activities, but extensive studies on this effect have not been conducted. In this context, our studies aimed to evaluate the antimicrobial properties of six raw bioproducts containing three classes of microbial polyketides biosynthesized by three *Monascus* strains through solid-state biosynthesis. As a methodology, we performed in silico predictions using programs such as PyMOL v3.0.4 and employed ESI-MS techniques to provide evidence of the presence of the six studied compounds in our bioproducts. The results obtained in silico were validated through in vitro studies using the Kirby-Bauer diffusion method on bacteria and fungi. The test performed in silico showed that Monascorubramine has the highest affinity for both Gram-positive and Gram-negative bacteria, followed by yellow polyketides such as Ankaflavin and Monascin. The estimated pharmacokinetic parameters indicated high gastrointestinal absorption and the potential to cross the blood-brain barrier for all studied compounds. However, the compounds also inhibit most enzymes involved in drug metabolism, presenting some level of toxicity. The best in vitro results were obtained for *S. aureus*, with an extract containing yellow *Monascus* polyketides. Predictions made for *E. coli* were validated in vitro for *P. aeruginosa*, *S. enterica*, and *S. marcescens*, as well as for fungi. Significant antibacterial properties were observed during this study for *C. albicans*, *S. aureus*, and fungal dermatophytes for crude bioproducts containing *Monascus* polyketides. In conclusion, the antimicrobial properties of *Monascus* polyketides were validated both in silico and in vitro. However, due to their potential toxicity, these bioproducts would be safer to use as topical formulations.

## 1. Introduction

Fungi of the *Monascus* genus are widely used in Asia for producing food products such as dairy and soy products [1,2,3,4], fermented vinegar [5], and alcoholic beverages made from cereals [6]. They are also used in traditional medicine, particularly in the form of food supplements like Beni Koji [7].

In Asia, Europe, and the USA, food supplements containing statins, obtained by fermenting rice with species such as *Monascus purpureus* or *Monascus pilosus* (commonly known as red yeast rice, or RYR) [7], are well known.

Red yeast rice (RYR) is extensively used in China for its dual role as food and medicine. As an ingredient, RYR is present in hundred foods and is believed to offer various health benefits, such as lowering blood lipid levels, boosting immunity, reducing blood pressure, and alleviating fatigue. RYR-based products are widely utilized. Additionally, RYR is a component of numerous Chinese herbal prescriptions and folk medicines, which have been documented in ancient texts and accredited by the China State Food and Drug Administration for treating conditions like hyperlipemia, fatigue, and diarrhea [8,9]. According to the studies by Zhu et al. performed in 2019 [8], RYR represents traditional Chinese medicine and food supplements, which are popular in East Asia, produced by fermenting cooked rice resulting in reddish purple kernels. The lovastatin content of RYR reduces blood cholesterol. Its poor oral bioavailability (<5%) is due to low solubility, extensive gut and liver metabolism, and P-glycoprotein efflux. The RYR extracts from the new formulations named LipoCol Forte, XZK, and Cholestin increase lovastatin’s absorption and dissolution rates, Human trials revealed that LipoCol Forte results in higher plasma concentration and faster absorption compared to lovastatin tablets. In a review by Chen et al. in 2023 [10], the variability of monacolin and citrinin content in RYR products, such as Xuezhikang, or those that contain RYR was highlighted. This analysis revealed potential safety concerns, such as the following: (1) variability in monacolin content, ranging between 0.15 and 3.37 mg/capsule; (2) average citrinin content of 228.3 μg per capsule. The studies performed by Vitiello et al. in 2023 [11] on several products with RYR from the Italian market reveal that the quality of marketed food supplements with RYR shows significant variation, particularly regarding the disintegration tests, which do not conform to pharmacopeial standards. This indicates that RYR manufacturing processes are not always validated or controlled.

However, a disadvantage of these food supplements, produced via fermentation with various *Monascus* species, is the inconsistency in the quality of the final product. This variability can pose risks for patients with chronic diseases, as it may interact with other medications and lead to serious poisoning [12]. In addition to statins, *Monascus* fungi produce compounds with antioxidant activity, such as dimerumic acid [13], as well as polyketides with immunomodulatory, antitumor [14], antibacterial [15,16,17,18,19], and antifungal properties. The antifungal activity is particularly effective against dermatophytes [20,21] and phytopathogens [22,23], and it is primarily attributed to the presence of polyketides. However, species of the *Monascus* genus also produce a mycotoxin called citrinin, which has toxic effects [24]. One especially interesting class of compounds from a therapeutic standpoint is polyketides. These can be colorless (such as lovastatin), red (Monascorubramine, Rubropunctamine, which contain carbon, hydrogen, and nitrogen), orange (Rubropunctatin, Monascorubrin), or yellow (Monascin, Ankaflavin) [25]. Analyses conducted using LC-ESI MS techniques have shown that *Monascus* fungi produce hundreds of metabolites. A distinctive feature of this fungi is that, depending on the conditions of biosynthesis and the composition of the culture medium, biosynthesis can be directed predominantly toward the production of red, orange, or yellow polyketides (pigments). The production of microbial polyketides is achieved through biosynthesis on a solid substrate [26], such as cereal grains, or through submerged biosynthesis using culture media containing carbon sources (sugars), organic or inorganic nitrogen sources, and macroelements and microelements [27]. The separation of colored polyketides can be performed using preparative chromatography [28] or selective extraction with organic solvents [20]. Their identification is best achieved through LC-ESI/MS techniques [29,30]. From an antimicrobial activity perspective, studies conducted in India demonstrated that *Monascus purpureus* exhibits an antagonistic effect on certain dermatophytes, such as *Microsporum* sp. and *Trichophyton* sp. [31]. When *Monascus purpureus* is cultivated in the presence of these dermatophytes, the growth of pathogenic microorganisms is inhibited by approximately 88–91% [31]. Regarding the analysis of polyketides produced by the *Monascus* species, the simplest method is the LC-MS technique. Analyses of the pigments produced by *Monascus* species, when using three types of cereal grains as culture media (i.e., rice, corn, and sorghum), showed that Rubropunctamine is the predominant compound in the final product, followed by Monapilol B, Xanthomonascin A, and nun, in descending order of concentration [32]. The solid substrate that yields the highest amount of Rubropunctamine consists of rice grains (87.3%), followed by sorghum grains (58.26%) and corn grains (57.03%) [32]. Regarding the antimicrobial activities of the pigments produced by *Monascus purpureus*, the best results are obtained with the crude extract obtained through total extraction in an alcoholic medium [31]. If the crude extract is fractionated to produce biopreparations enriched in red, orange, or yellow pigments, the best activity is observed with the biopreparation containing yellow pigments, although the results are still lower compared to those of the crude extract [20,31]. These bioproducts exhibit activity against microorganisms such as *S. aureus*, *E. coli*, *P. aeruginosa*, *Bacillus* sp., *Klebsiella* sp., and *Proteus* sp. The studies performed in vitro regarding the photoprotective effects of the pigments produced by *Monascus purpureus* on a standardized HaCaT cell line showed that they can inhibit the cell destruction caused by ultraviolet radiation exposure [33]. The literature also reports that pigments produced by *Monascus* species have antimicrobial effects on phytopathogen microorganisms such as *P. expansum*, *R. stolonifer*, and *A. niger* [34]. The antimicrobial effect of these pigments is attributed to the disruption of the integrity of the cytoplasmic membrane in phytopathogenic fungi [34].

In silico tests regarding the antimicrobial activity of some polyketides produced by *Monascus* sp. or derived from the compounds biosynthesized by them are not currently available. The most common in silico studies are those related to antidiabetic activity, anti-cholesterol activity [35,36], anti-cardiovascular or antitumor disease [37,38]. Regarding antidiabetic activity, the in silico tests showed that among the pigments produced by *Monascus* sp., there is a potential candidate with antidiabetic properties—namely a derivative of Glycyl Rubropunctatin [35], which can be obtained under certain biosynthesis conditions. The tests regarding the anti-cholesterol-lowering and antitumor properties by Singgih et al. [36] were performed on 14 derivatives of Monacolin and 33 pigments derived from *Monascus*. These studies showed that the Gibbs Free Energy corresponding to the complex formed between each of the 47 ligands and each binding site was, in each case, higher than the value obtained for the chosen references specific to the two studied docking sites. Moreover, the toxicity studies carried out in silico [39] on 57 pigments produced by *Monascus* showed that all of these compounds have mutagenic potential. Additionally, the predictive model used indicated liver toxicity for all 57 pigments. In each of these cases, the results were different and were due to the limitations given by the prediction program used.

The present studies were carried out with three classes of polyketides, obtained by selective solvents extraction from bioproducts produced by solid state biosynthesis from three *Monascus* species: two standardized *Monascus* species from recognized international collections (DSM 1379; MUCL 28962) and a highly productive *Monascus* strain obtained by irradiation in a field of accelerated electrons of the strain MUCL 28962).

This study aims to draw a parallel between the antimicrobial activity of the three classes of polyketides isolated from the three *Monascus* species and to evaluate their antimicrobial activity in silico. The toxicity and pharmacological tests performed in silico aimed to establish the best model of administration for a certain class of polyketides (oral administration, IP administration, topical administration) to increase their efficiency and ensure their safe use. Predictions regarding the toxicity of these compounds represent an important element because they could elucidate the adverse effects recorded and reported by medical case studies, particularly since the data reported so far are insufficient or were generated with other types of prediction programs.

**Figure 1:**
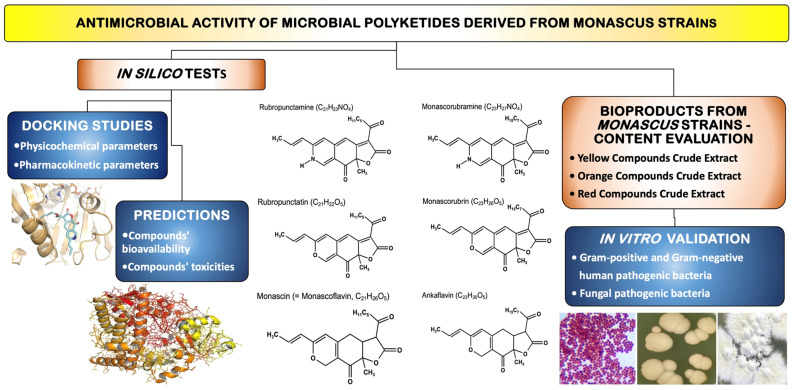
Experimental study design.

The main objective of this research is to highlight the antimicrobial effects of eight microbial polyketides (pigments) contained in the biomass obtained by biosynthesis on a solid substrate (rice) using three species of Monascus. To achieve this, the following studies and tests were conducted:

- In silico studies regarding the molecular docking of six compounds found in the pigments produced by *Monascus*, targeting three models of pathogenic microorganisms—*S. aureus* (G+ model), *E. coli* (G− model), and *C. albicans* (fungal model);
- In silico evaluation of the physicochemical and pharmacokinetic parameters of the eight compounds;
- Predictions regarding the bioavailability and toxicity of the six compounds;
- Evaluation of the relative composition of three bioproducts (i.e., red yeast rice RYR) obtained from *Monascus purpureus*, *Monascus ruber*, and a highly productive strain of *Monascus ruber*;
- In vitro studies regarding the activity of bioproducts containing yellow, orange, and red pigments obtained by biosynthesis on a solid substrate with three strains of *Monascus*. The studies in vitro were conducted on pathogenic microorganisms, including G+ bacteria, G− bacteria, and fungi. The experimental study design is illustrated in Figure 1.

## 2. Results

### 2.1. In Silico Predictions

The studies were conducted in silico for six chemical compounds—Monascin, Ankaflavin (yellow polyketides), Rubropunctatin, Monascorubrin (orange polyketides), Monascorubramine, and Rubropunctamine (red polyketides) (referred to as ligands) considered as the major pigments—and showed the following:

The free energy required to dock the studied ligands in the human dihydrofolate reductase (DHFR) situs is much lower compared to the free energy needed to dock in the studied microbial DHFR sites. Analyzing the selectivity index value obtained for each microbial target reveals that all values are less than one (<1) indicating that the studied ligands exhibit selectivity for human DHFR (Table 1).

For *S. aureus* (a model for Gram-positive bacteria), the docking energy of Monascorubramine (ΔG = −8.969 kcal/mol) is significantly lower than that of Trimethoprim (ΔG = −8.584 kcal/mol). Comparable docking energies to Trimethoprim were obtained for Rubropunctamine (ΔG = −8.450 kcal/mol), Monascorubrin (ΔG = −8.302 kcal/mol), and Monascin (ΔG = −8.082 kcal/mol). The dissociation constant (Kd) values indicate that the most stable complex is formed with Monascorubramine (Kd = 0.266 µM), followed by Trimethoprim (Kd = 0.51 µM), Rubropunctamine (Kd = 0.640 µM), and Monascorubrin (Kd = 0.82 µM). This suggests that the two red polyketides and one orange compound will preferentially dock in the binding site of *S. aureus*, as lower ΔG and Kd values are more favorable for these polyketides.

In the case of *E. coli* (a model for Gram-negative bacteria), most ligands exhibit lower free docking energies than Trimethoprim, with the exceptions of Rubropunctamine and Monascin, which require higher energies (see Table 1). Monascorubramine binds with the least energy in the docking pose (ΔG = −8.537 kcal/mol) and shows a lower dissociation constant (Kd = 0.552 µM), followed by Ankaflavin (ΔG = −8.499 kcal/mol), Monascorubrin (ΔG = −8.424 kcal/mol), and Rubropunctatin (ΔG = −8.303 kcal/mol). These findings suggest that Monascorubramine, Ankaflavin, Monascorubrin, and Rubropunctatin are the preferred ligands for docking at the ecDHFR binding site in *E. coli*.

**Figure 2:**
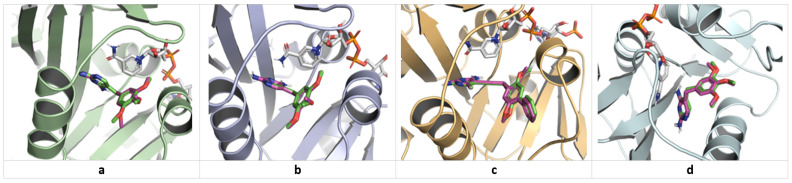
Molecular docking validation—superposition of predicted poses (pink) of co-crystallized inhibitors on initial conformations (green): (**a**) trimethoprim in saDHFR binding site (PDB ID: 2w9s, RMSD 0.6535 Å); (**b**) trimethoprim in ecDHFR binding site (PDB ID: 7mym, RMSD 0.3521 Å); (**c**) UCP11E in caDHFR binding site (PDB ID: 4hoe, RMSD 0.4389 Å); (**d**) trimethoprim in hDHFR binding site (PDB ID: 2w3a, RMSD 0.9559 Å).

**Figure 3:**
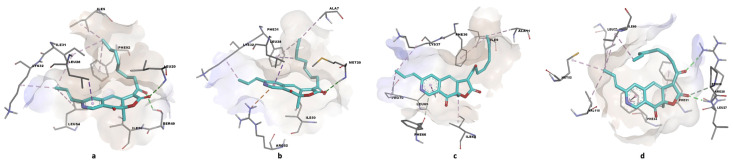
Predicted binding poses of Monascorubramine in DHFR active sites. (**a**) saDHFR; (**b**) ecDHFR; (**c**) caDHFR; (**d**) hDHFR.

**Figure 4:**
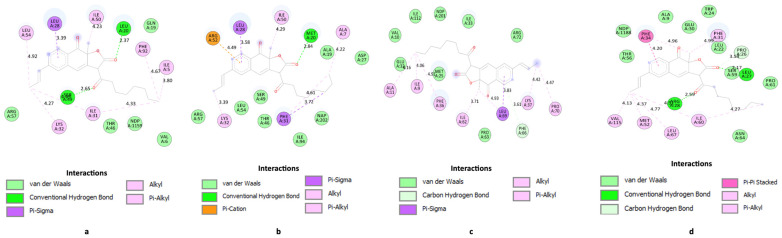
2D diagrams of predicted molecular interactions between Monascorubramine and active sites of DHFR homologues. (**a**) saDHFR; (**b**) ecDHFR; (**c**) caDHFR; (**d**) hDHFR.

Regarding *C. albicans* (a model for fungi), the studied ligands exhibit higher free energies for ligand-binding site complex formation compared to UCP11E. However, two relatively stable complexes (Kd < 1 µM) are formed, with Rubropunctatin being the most stable (Kd = 0.720 µM), followed by Monascin (Kd = 0.763 µM). Overall, Rubropunctatin shows an affinity for all binding sites. Figure 2, Figure 3 and Figure 4 illustrate the docking of trimethoprim (co-crystallized) and Monascorubramine, along with their interactions in the microbial/human DHFR docking sites.

The physicochemical properties predicted for the studied ligands (Table 2 and Table 3) show similarities in terms of the number of acceptor and donor hydrogen bonds as well as the number of violations of Lipinski’s Rule of Five (without violation of this rule for the studied ligands). The octanol–water partition coefficient (WlogP) values for the studied ligands are also close, ranging from 3.51 to 4.41.

**Figure 5:**
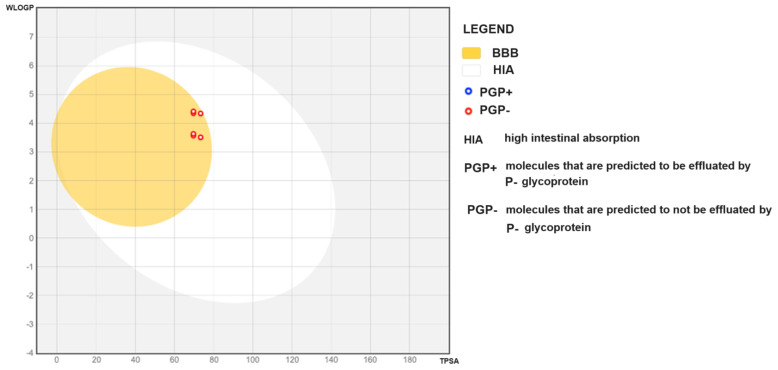
“Boiled egg” diagram illustrating the distribution of the investigated compounds in the chemical space of molecules that are absorbed in the gastrointestinal (GI) tract or passively permeate the blood–brain barrier (BBB) based on calculated WlogP (octanol/water partition coefficient) and TPSA (topological polar surface area) values. Molecules located in the “egg yolk” are predicted to passively permeate through the BBB. Molecules located in the white area are predicted to be passively absorbed in the GI tract.

Regarding solubility, based on the values predicted by the model, the ligands fall into the categories of “moderately soluble” and “soluble”. The bioavailability coefficient is 0.85 for the yellow and orange polyketides. All the studied compounds exhibit high intestinal absorption (HIA) and can penetrate the blood–brain barrier (BBB) (Figure 5).

The predictions regarding the pharmacokinetic parameters for the six ligands indicate that, in general, they have the potential to inhibit the Cytochrome P450 (CYP) enzyme complex, which is involved in the metabolism of drugs and xenobiotics (Table 3). This suggests that the studied molecules may have the potential to interact with the metabolism of other substrates of CYP isoforms (Table 4).

The predictive model indicates a lack of hepatotoxicity, neurotoxicity, and mutagenicity for all studied ligands. However, all ligands studied show BBB (blood–brain barrier) toxicity (Table 4) and respiratory toxicity (*p* ≥ 0.79). For all yellow and red polyketides studied, the model also indicates immunotoxicity. Notably, the model predicts that none of the compounds studied are effluated by P-glycoprotein.

According to the model predictions, Monascin and Ankaflavin fall into toxicity class 3 (LD_50_ = 250 mg/kg body weight), as do the orange polyketides Monascorubrin and Rubropunctatin (LD_50_ = 130 mg/kg body weight).

The red polyketides fall into toxicity class 4, with predicted LD_50_ values of 416 mg/kg body weight for Monascorubramine and 2000 mg/kg body weight for Rubropunctamine. Based on these data, the toxicity of the studied compounds appears to increase in the following order:

Rubropunctatin and/or Monascorubrin > Ankaflavin and/or Monascin > Monascorubramine > Rubropunctamine.

Overall, the orange polyketides demonstrate higher toxicity compared to the yellow or red polyketides studied.

### 2.2. Analysis of Bioproducts Obtained from Monascus sp.

**Figure 6:**
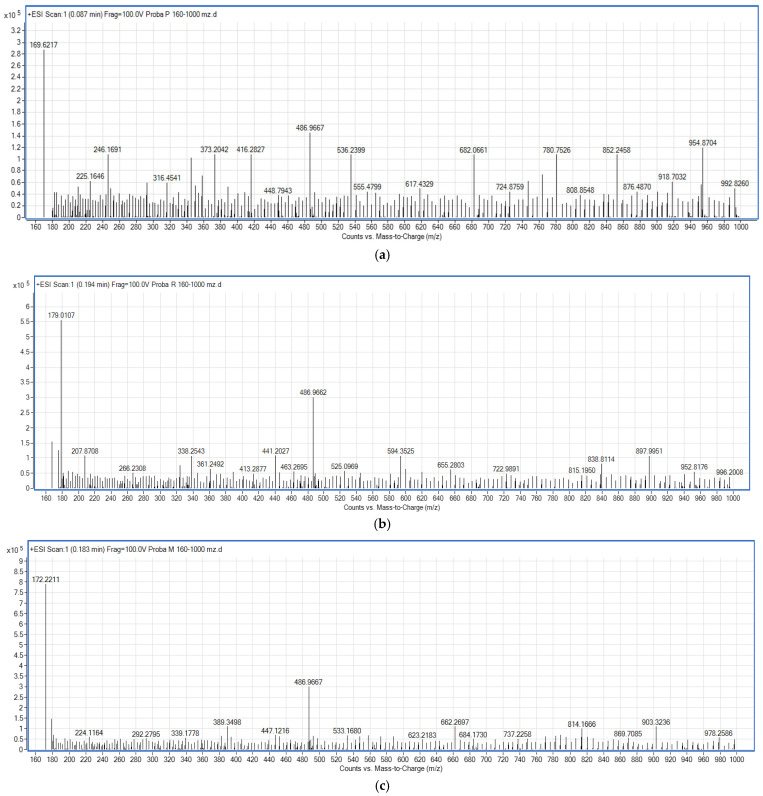
ESI-MS analysis of a total alcoholic extract of the following: (**a**) *Monascus purpureus*; (**b**) *Monascus ruber*; (**c**) *Monascus* sp. 3 *(Monascus ruber*; highly productive).

The qualitative analysis of the data obtained from the ESI-MS analysis allowed for the assignment of only a limited number of compounds (Figure 6a–c and Table 5). In the extract obtained from the bioproduct resulting from *M. purpureus*, all the studied compounds were highlighted. On the other hand, the extract obtained from *M. ruber* does not contain Ankaflavine and Monascorubrine. In the extract obtained from the highly productive *M. ruber*, the compound Ankaflavin appears, but the compound Monascorubrine is still absent. A relatively high content of Monascorubramine was found in the extract obtained from the bioproduct derived from the highly productive *M. ruber* strain.

### 2.3. Results Validation Through In Vitro Tests

**Figure 7:**
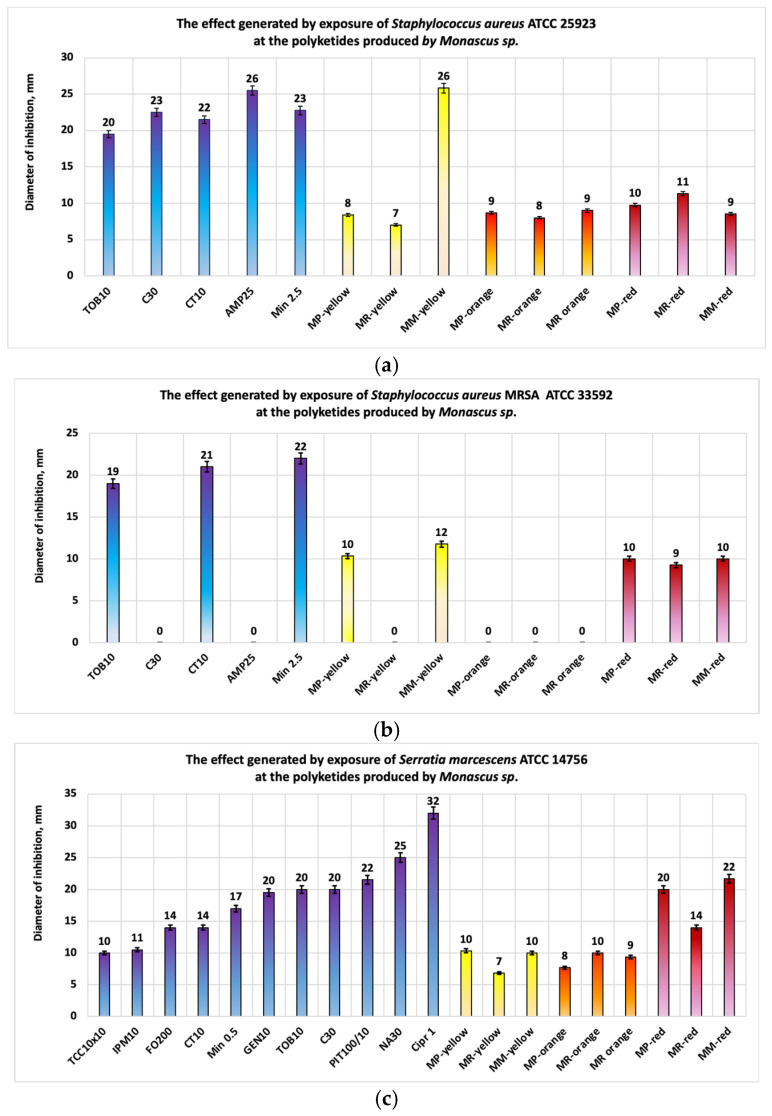

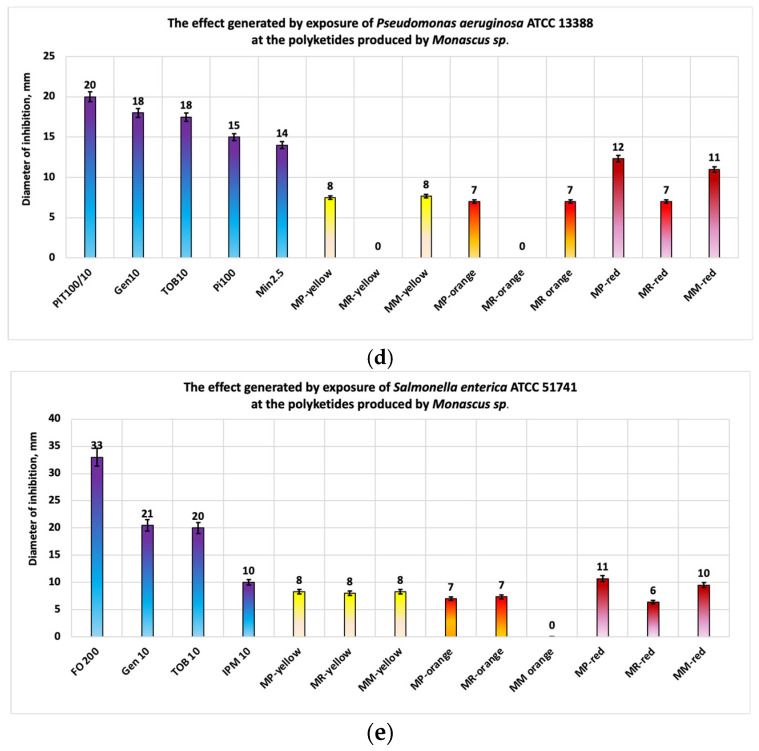
Antibacterial properties of polyketides obtained from Monascus-derived bioproducts: (**a**) antibacterial properties for *S. aureus* (yellow polyketides exhibit the best activities); (**b**) antibacterial properties for *S. aureus* MRSA (yellow polyketides exhibit moderate activities); (**c**) antibacterial properties for *S. marcescens* (red polyketides exhibit the best activities); (**d**) antibacterial properties for *P. aeruginosa* (red polyketides exhibit moderate antimicrobial activities); (**e**) antibacterial properties for *S. enterica* (red polyketides exhibit local-moderate antimicrobial activities).

The studies conducted on *S. aureus* (Figure 7a) showed that the largest inhibition diameter was obtained with the bioproduct MM-yellow (inhibition diameter = 26 mm), which contains yellow polyketides. This effect surpasses that of the antibiotic Minocycline and is comparable to that of Ampicillin. The other extracts exhibit a local antimicrobial effect, with bioproducts containing red polyketides (including Monascorubramine) standing out. In the case of *S. aureus* MRSA, the antimicrobial effect is both local and moderate (Figure 7b). Among the inhibition diameters observed, the highest value was obtained for MM-yellow (inhibition diameter = 12 mm), followed by MP-yellow (inhibition diameter = 10 mm) and the extracts containing red polyketides. Extracts containing orange polyketides did not show antimicrobial activity against *S. aureus* MRSA.

Regarding Gram-negative bacteria, the largest inhibition diameters for all studied cases (*S. enterica*, *P. aeruginosa*, *S. marcescens*) were obtained from the extracts containing red polyketides (Figure 7c–e) biosynthesized by highly productive *M. ruber* (*MM-red*) and *M. purpuresus* (*MP-red*).

**Figure 8:**
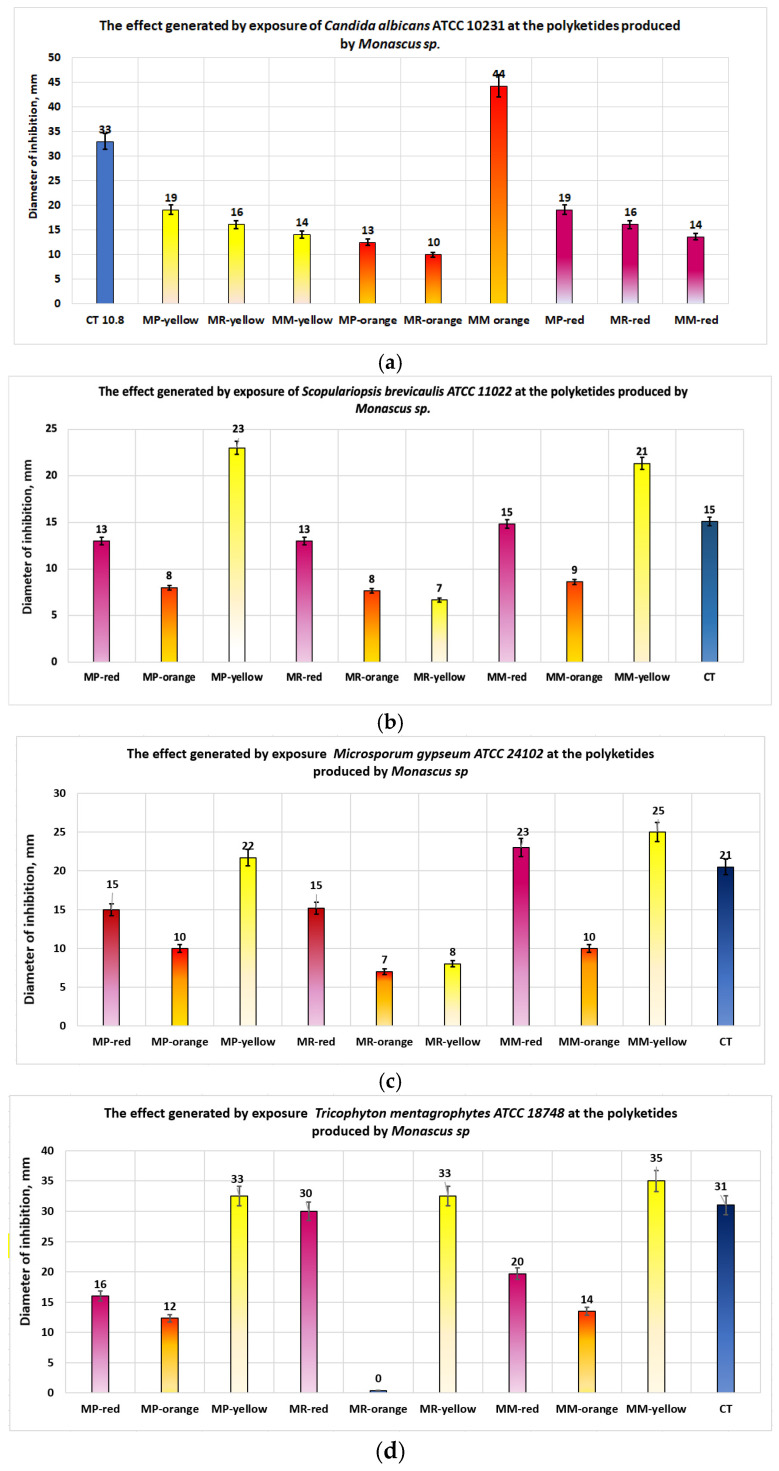
Antifungal properties of polyketides obtained from Monascus-derived bioproducts for the following: (**a**) *Candida albicans*; (**b**) *S. brevicaulis*, (**c**) *M. gypseum*; (**d**) *T. mentagrophytes*.

In the case of *S. marcescens*, these extracts demonstrated a significant antimicrobial effect, with the best results obtained from the bioproducts MM-red (inhibition diameter = 22 mm) and MP-red (inhibition diameter = 20 mm). The experimental studies conducted on fungi (Figure 8a–d) showed that for *C. albicans*, the fraction containing orange polyketides synthesized by highly productive *Monascus ruber* exhibited distinctly significant antimicrobial activity, with an average inhibition diameter of 44 mm—a value superior to all standardized antibiotics used for comparison. In descending order of the average inhibition diameters are the bioproducts MP-red and MP-yellow (inhibition diameter = 19 mm), followed by MR-red and MR-yellow (inhibition diameter = 16 mm) and MM-red and MM-yellow (inhibition diameter = 14 mm) (Figure 8a).

For dermatophytes (Figure 8b–d), the best results were generally obtained from the fractions containing yellow polyketides from bioproduct MM-yellow, with average inhibition diameters of 21 mm for *S. brevicaulis*, 25 mm for *M. gypsum*, and 35 mm for *T. mentagrophytes*. The next best results were obtained from the bioproduct MP-yellow, which exhibited inhibition diameters ranging from 22 mm to 33 mm.

## 3. Discussions

The results of the in silico study confirm the in vitro findings, which indicate that yellow and red polyketides have promising antimicrobial activity against *S. aureus*, including on resistant strains (Figure 7a,b), aligning with data reported in the literature [13,15,16]. Yellow polyketides exhibit greater bioavailability (score of 0.85) compared to Monascorubramine (score of 0.55). However, both types of compounds are effective, with red polyketides being more selective for the DHFR enzyme specific to *S. aureus* (Table 1).

Monascorubramine (a red polyketide) binds through van der Waals interactions and hydrogen bonds (Figure 4a) in the docking site. This compound displays high selectivity for the microbial DHFR enzyme but inhibits most of the CYP enzymes (Table 3) involved in drug metabolization.

In contrast, yellow polyketides inhibit a maximum of three out of the five enzymes analyzed (Table 3). Due to their toxicity (Table 4) and the potential to cross the blood-brain barrier (Figure 5), it is recommended to incorporate these compounds into topical products aimed at *S. aureus* or *S. aureus* MRSA infections, where yellow polyketides provide significant activity and Monascorubramine offers moderate activity. The toxicity of these compounds was anticipated throughout the in silico studies conducted to evaluate the possibility of using polyketides biosynthesized by microorganisms from the *Monascus* genus for treating diabetes and cardiovascular diseases and for their antitumor effects [35,36,37]. During these studies, mutagenic effects and hepatic toxicities were anticipated [38].

The results obtained in silico using a Gram-negative microorganism model (i.e., *E. coli*) showed that the favored compounds for docking are Monascorubramine (with the lowest docking energy), Ankaflavin, Monascorubrine and Rubropunctatine (Table 1), (all these ligands form the most stable complex with the docking site). The results obtained in vitro on several types of Gram-negative microorganisms (Figure 7c–e) confirm that the red polyketides produce large inhibition diameters against microorganisms such as *S. enterica*, *P. aeruginosa*, and *S. marcescens*, with these results being in agreement with data reported by other researchers [13,15]. Additionally, the presence of Monascorubramine was detected in all obtained bioproducts (Table 5).

Regarding the docking mode in the selected microbial situs, it occurs primarily through van der Waals interactions (Figure 4b). Here as well, due to the pronounced toxicity (Table 3 and Table 4, Figure 5) also anticipated by previous in silico studies [35,36,37,38], it is recommended that fractions containing red polyketides be incorporated into topical formulations. 

Regarding the in silico predicted effects for fungi (*C. albicans* model), it was observed that the docking energies in the proposed microbial pose for all compounds are higher than those of the selected reference (Table 1). Both Rubropunctatin (an orange polyketide) and Monascin (a yellow polyketide) have a dissociation constant of less than one (<1) and form a more stable complex with the docking site. So, the complex formed between the ligand and the docking site is more stable. Judging this way, the most stable complex is the one formed between the docking site and Rubropunctamin (Kd = 1.694), followed by the complex formed with Monascorubramine (Kd = 1.493), Monascorubrin (Kd = 1.285), and Ankaflavin (Kd = 1.232).

The docking between the ligand and the docking site specific to *C. albicans* is predominantly formed through van der Waals interactions (Figure 4c). In this case, red polyketides appear to be the favorites, followed by orange and yellow ones. The in vitro results for *C. albicans* (Figure 8a) show that the best results are obtained for the bioproducts containing orange polyketides, derived from the highly productive *Monascus* strain, where the concentration of crude extract with orange polyketides is approximately 4800 mg/L, (making it the most concentrated polyketide-enriched extract). Monascorubrin, the preferred ligand in this case, has a bioavailability coefficient close to the unit (0.85, Table 2). However, it inhibits all five hepatic enzymes in the CYP complex (Table 3), may cause respiratory cytotoxicity, and is classified in the toxicity class 3. Moreover, the orange polyketides proposed in this study show the highest toxicity among the compounds studied (DL50 = 130 mg/kg body). The best activity against *C. albicans* is exhibited by orange polyketides due to their high concentration in the crude extract on one hand and their lower docking energy compared to other *Monascus* sp.-derived compounds (ΔG = 8.38) on the other. The results obtained for dermatophytes show that yellow polyketides are more active against these microorganisms, followed by the red ones (Figure 8b–d), with the findings aligning with previous studies [31]. Toxicity issues also arise in this context, and the best formulations are topical ones, as they help reduce the product’s toxicity.

The ESI-MS analysis of the bioproducts obtained through biosynthesis with three types of Monascus demonstrated that each brut bioproduct contains at least five of the six compounds analyzed in silico. Regarding the predicted toxicity of these six types of polyketides, it can be concluded that these findings may help explain the recent controversy surrounding the product called Beni Koji [40], produced by Kobayashi Pharmaceutical in Japan [32]. In this case, five individuals died, and approximately 200 patients were hospitalized due to renal dysfunction caused by yeast red rice (fermented with *Monascus pilosus*) consumed as a treatment for hyperlipidemia [7,40,41].

Although the causes are unknown, it is highly likely that the enzymes involved in metabolizing the active substances from red rice were inhibited by the six polyketides analyzed in this study. The result was likely an accumulation of toxins in the body; since the polyketides from Beni Koji are soluble, they are absorbed in the gastrointestinal tract and can cross the blood–brain barrier.

## 4. Materials and Method

### 4.1. In Silico Studies

#### 4.1.1. Docking Predictions

Molecular docking simulations were carried out following the approach detailed in our previously published work. The predicted binding poses were analyzed using PyMOL v3.0.4 (The PyMOL Molecular Graphics System, Schrödinger, LLC, New York, NY, USA), and 2D interaction diagrams were generated with BIOVIA Discovery Studio Visualizer v17.2.0 (BIOVIA, Dassault Systèmes, 2016, San Diego, CA, USA) [42,43]. Specific dihydrofolate reductases from *S. aureus*, *E. coli*, and *C. albicans* as well as human dihydrofolate reductase were selected as molecular targets.

The following PDB codifications were used:

- 2w9s—*S. aureus* dihydrofolate reductase (saDHFR), reference: trimethoprim (5-(3,4,5-trimethoxybenzyl)pyrimidine-2,4-diamine) [44];
- 7mym—*E. coli* dihydrofolate reductase (ecDHFR), reference: trimethoprim [45];
- 4hoe—*C. albicans* dihydrofolate reductase (caDHFR), reference: UCP11E (5-[3-(2,5-dimethoxy-4-phenylphenyl)but-1-yn-1-yl]-6 methyl pyrimidine-2,4-diamine) [45];
- 2w3a—human dihydrofolate reductase (hDHFR), reference: trimethoprim [45];

#### 4.1.2. Prediction of ADMET Properties

The physicochemical and ADMET (absorption, distribution, metabolism, excretion, toxicity) properties of the identified fungal metabolites were predicted using SwissADME [46] and ProTox 3.0, respectively [47,48].

For the studies performed in silico regarding the antimicrobial activity on Gram-negative and Gram-positive bacteria, the trimethoprim compound was chosen as the reference substance (Co-crystallizate) because it has activity on a wide spectrum of Gram-negative and Gram-positive bacteria, representing a referential good for both types of bacteria. Moreover, the mechanism of action for trimethoprim is known: specifically, trimethoprim inhibits bacterial dihydrofolate reductase, an essential enzyme in folic acid synthesis, which is crucial for DNA and RNA synthesis [49,50,51]. Additionally, trimethoprim has a known safety profile, as demonstrated by data obtained in clinical studies comparing it with a compound with proven efficiency and a demonstrated safety profile [52,53].

The molecular docking study was performed to predict the inhibitory activity of six compounds against microbial dihydrofolate reductases (DHFRs): Monascin and Ankaflavin (yellow polyketides), Rubropunctatin and Monascorubrin (orange polyketides), Monascorubramine, and Rubropunctamine (red polyketides). These compounds, referred to as ligands, are considered the major pigments. We chose DHFR as a pharmacological target due to its key role in DNA synthesis, making it an attractive target for the discovery of antimicrobial agents. Additionally, human DHFR was included in the screening campaign to calculate selectivity indices against the human homologue. Even though the prediction program may give low selectivity indices against hDHFR (limiting the therapeutic application in infectious diseases due to potential cytotoxicity), the studied compounds could represent potential antitumor agents by directly assessing antiproliferative effects due to DHFR inhibition of cancer cells.

### 4.2. Microorganisms Used

(a) Three *Monascus* strains were used to obtain RYR: *Monascus purpureus* DSM 1379, *Monascus ruber* MUCL 28962, and a highly productive *Monascus ruber* strain. The last strain was received as a gift from Prof. Octavian Duliu of the Department of Atomic and Nuclear Physics at the University of Bucharest, Romania.
(b) Bacteria: *Staphylococcus aureus* ATCC 25923, *Staphylococcus aureus* MRSA ATCC 33592, *Serratia marcescens* ATCC 14756, *Pseudomonas aeruginosa* ATCC 13388, and *Salmonella enterica* ATCC 51741.
(c) Fungi: *Candida albicans* ATCC 10231, *Microsporum canis* ATCC 10214, *Trichophyton mentagrophytes* ATCC 18748, *Microsporum gypseum* ATCC 24102, and *Scopulariopsis brevicaulis* ATCC 1102.

### 4.3. Monascus Bioproducts

The three bioproducts (red yeast rice, RYR) were obtained following the methodology presented by Agboyibor et al. [40]. For this purpose, 100 g of rice was added to a 1 L Erlenmeyer flask, which was autoclaved for 20 min at 115 °C. After sterilization and cooling, 10 mL of inoculum containing 10^7^ spores/mL was aseptically added to the Erlenmeyer flask.

The spore inoculum was previously prepared from fresh strains, cultivated by growing each *Monascus* species for 7 days in an incubator (LbX Instruments, Barcelona, Spain) at 25 °C. Sterile Petri plates were inoculated with each *Monascus* strain on potato dextrose agar in a Bio48 Faster microbiological hood (Cornaredo, Italy).

The spores produced after 7 days were collected from the surface of each mycelium using sterile distilled water. The number of spores in the resulting suspension was determined using a hemacytometer, and their concentration was adjusted up to the specified value with sterile distilled water.

The flask was sealed with sterile cellulose plugs and incubated at 37 °C for 2 weeks. After incubation, the red rice produced was sterilized at 60 °C for 4 h. Once cooled, the RYR obtained was ground and stored in the dark at room temperature.

In this way, three types of bioproducts were obtained, corresponding to the three *Monascus* strains. Microbial extracts containing primarily yellow, orange, and red polyketides were subsequently obtained from these bioproducts by extraction with selective solvents.

### 4.4. Extracts with Yellow, Orange, and Red Monascus Polyketides

The extraction of yellow polyketides, orange polyketides, and red polyketides was carried out according to the methodology presented by Majhi et al. [20].

Each solvent used in the selective extraction was subjected to evaporation after extraction at 50 °C using a rotary evaporator (Heidolph, Schwabach, Germany). Each crude extract obtained was weighed and dissolved in 100 mL DMSO. The final crude extract concentrations in each case are presented in Table 6.

### 4.5. Analysis of RYR Bioproduct

The LC-MS analysis was conducted using an Agilent Technology LC-MS TOF 6224 device (Santa Clara, CA, USA) with operating parameters established by Liang et al. [30].

(a) Obtaining Raw Extracts

To obtain the raw extract, a 0.3 g sample of each red yeast rice (RYR) was extracted with 10 mL of 75% ethanol in an ultrasonic bath for 30 min. The extraction process was repeated three times, each with 10 mL of solvent. The resulting solution was then centrifuged at 13,000 rpm. The obtained supernatant was subjected to solvent removal at 50 °C using a rotavapor (Heidolph Instruments GmbH & Co., Schwabach, Germany) to obtain the crude extract, which contains all compounds. This crude extract was then dissolved in 10 mL ethanol and subjected to the LC-MS analysis.

Only this extract allowed for the qualitative detection of the analyzed compounds. Because the pure compounds studied in our work are not yet commercially available, the concentration of each compound determined by the LC-MS was relative.

(b) Obtaining Extracts Enriched with Yellow, Orange, or Red Polyketides

**Figure 9:**
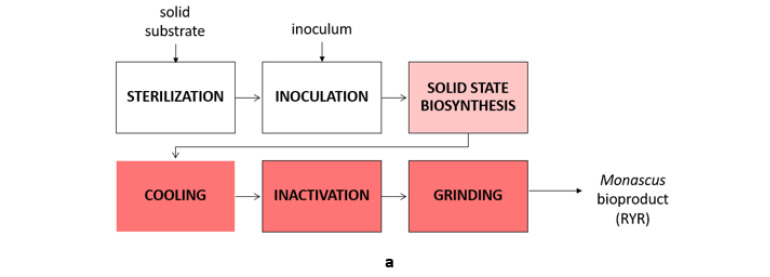

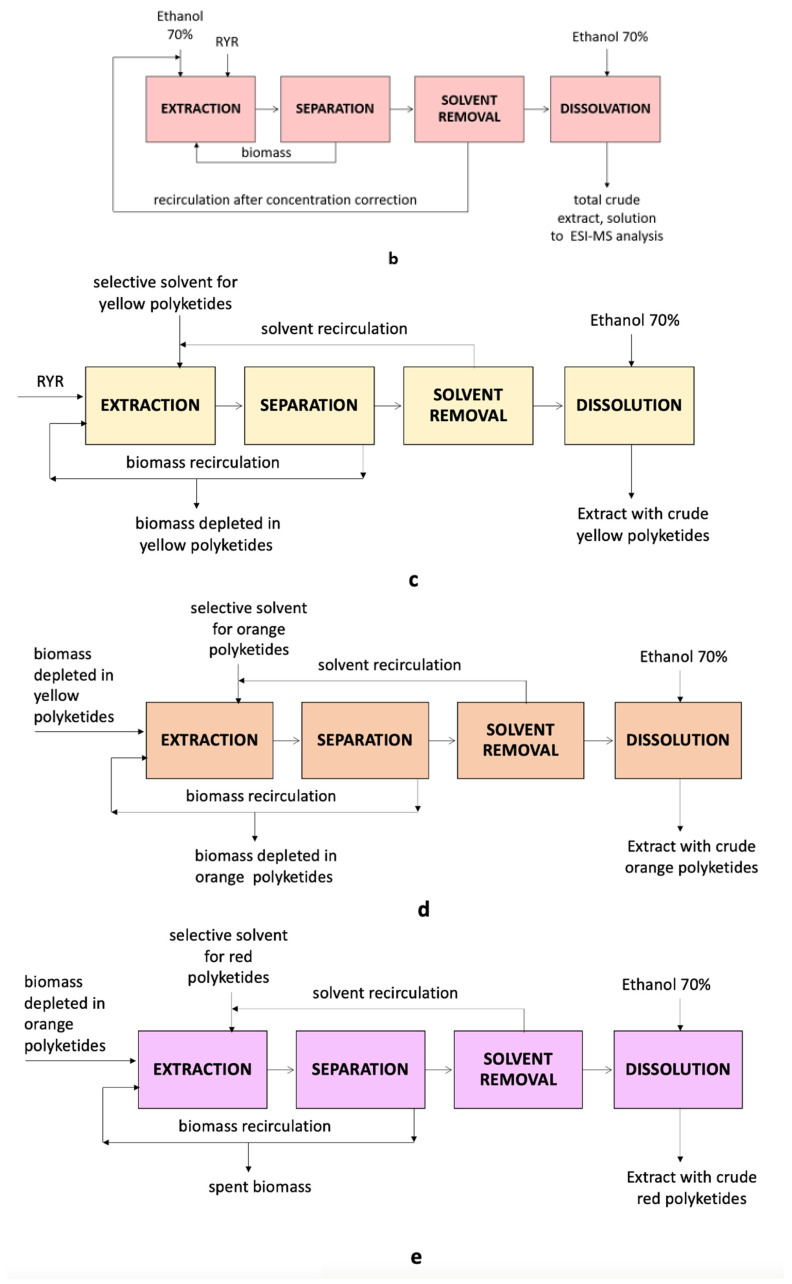
Flow diagram used to obtain enhanced extracts of yellow, orange, and red polyketides: (**a**) Solid-state biosynthesis of *Monascus* bioproducts (RYR); (**b**) Sample preparation of *Monascus* bioproducts for analysis; (**c**) Obtaining *Monascus* extract with yellow polyketides; (**d**) Obtaining *Monascus* extract with orange polyketides; (**e**) Obtaining *Monascus* extract with red polyketides.

In order to obtain polyketide-enriched extracts, 0.3 g of each solid RYR bioproduct was extracted with 10 mL of a solvent specific to each class of polyketides. This procedure was repeated at least three times with the same amount of biomass or until the extraction solvent was no longer colored. A flow diagram illustrating the extraction process for obtaining crude extracts enriched with yellow, orange, or red polyketides is shown in Figure 9. The crude extracts, obtained after removing the selective extraction solvent, were weighed and dissolved in 10 mL of 70% ethanol.

The analysis of the crude extracts, which contain yellow, orange, and red polyketides obtained with selective solvents, was attempted using ESI-MS techniques, with parameters established by Liang et al. [30]. However, in the ionization chamber, the molecules fragmented, and the resulting fragments could not be adequately analyzed in the absence of commercial standards. A 2023 prospective study found that reagent companies currently market only two compounds.

### 4.6. In Vitro Validation Tests

Antimicrobial tests on bacteria and fungi were conducted according to the methodologies presented by Zaharie et al. [54] for fungi and according to the methodologies presented by Babeanu et al. [55] for bacteria with the same devices, raw materials, and methods [54,55]. The antimicrobial activity was evaluated by comparing the resulting inhibition diameters with those obtained in the case of the use of diffusive discs impregnated with antibiotics. For this purpose, diffusive disks of 6 mm diameter, impregnated with antibiotics (BioRad Lab, Hercules, CA, USA), were used.

### 4.7. Statistical Analysis

All antimicrobial tests were performed in triplicate, and the results are presented as average values ± standard deviation.

## 5. Conclusions

Docking studies conducted in silico with six major compounds biosynthesized by *Monascus* sp.—namely Rubropunctamine, Monascorubramine, Rubropunctatin, Monascorubrin, Monascin, and Ankaflavin—targeted the dihydrofolate reductase specific to three microorganisms (*S. aureus*, *E. coli*, and *C. albicans*). These studies showed that the docking energy required to replace the referential choice (trimethoprim or UCP11E) was comparable to, or in some cases lower than, that of the natural ligand (Monascorubramine). Theoretically, all the compounds studied have the potential to exhibit antimicrobial activity. The tests conducted in vitro were undertaken with three types of crude extracts enriched in yellow, orange, and red polyketides obtained by biosynthesis in the solid phase using three strains of *Monascus* (two standardized strains and one highly productive strain). The results obtained in vitro confirmed the in silico predictions for *S. aureus* and *S. aureus* MRSA. The best results were achieved with a bioproduct derived from a highly productive *Monascus* strain, which contains a higher crude extract concentration enriched in yellow polyketides. The results obtained in silico for *E. coli* suggest that these findings could be extended to other Gram-negative bacteria, such as *P. aeruginosa*, *S. marcescens*, and *S. enterica*—a fact supported by the results obtained in vitro. In this context, all crude extracts enriched in red polyketides exhibited moderate antimicrobial activity against the tested Gram-negative bacterial strains. For *C. albicans*, the results in silico indicate a preference for red and orange polyketides. The in vitro results further support this finding, revealing that the bioproduct obtained from a highly productive *Monascus* strain, which contains a crude extract rich in orange polyketides, yielded excellent results, obtaining an average inhibition diameter of 44 mm—a value greater than the inhibition diameter obtained for all the standardized antibiotic reagents used. This effectiveness may be due to *C. albicans* behaving more like yeast than a fungus. In contrast, for dermatophytes (true fungi), the in silico results aligned with the in vitro findings, showing significant antimicrobial activity from yellow polyketides. In this case, antimicrobial activity obtained in vitro was comparable to or greater than that of clotrimazole. The most effective bioproducts, which contain crude extract enriched in yellow polyketides, were those obtained from a highly productive *Monascus* strain and a parental *Monascus* strain. Regarding physicochemical and pharmacological predictions, assessments indicated that all six compounds could be absorbed from the gastrointestinal tract and have the potential to penetrate the blood–brain barrier. However, pharmacokinetic evaluations suggest that most of these compounds may inhibit enzymes involved in their metabolization, which could lead to accumulation in the body. Therefore, these bioproducts are recommended for developing formulations with antimicrobial activity intended for topical administration.

## Figures and Tables

**Table 1:** Molecular docking results on DHFR.

	saDHFR			ecDHFR			caDHFR			hDHFR	
Compound	ΔG (kcal/mol)	Kd (μM)	SI	ΔG (kcal/mol)	Kd (μM)	SI	ΔG (kcal/mol)	Kd (μM)	SI	ΔG (kcal/mol)	Kd (μM)
Ankaflavin	−7.920	1.565	0.111	−8.499	0.589	0.296	−8.062	1.232	0.142	−9.220	0.174
Monascin	−8.082	1.191	0.133	−8.162	1.040	0.152	−8.346	0.763	0.207	−9.278	0.158
Monascorubramine	−8.969	0.266	0.928	−8.537	0.552	0.448	−7.948	1.493	0.166	−9.013	0.247
Monascorubrin	−8.302	0.821	0.753	−8.424	0.669	0.925	−8.037	1.285	0.482	−8.470	0.619
Rubropunctamine	−8.450	0.640	0.536	−7.547	2.937	0.117	−7.873	1.694	0.202	−8.820	0.343
Rubropunctatin	−7.826	1.834	0.256	−8.303	0.820	0.573	−8.380	0.720	0.652	−8.633	0.470
Trimethoprim	−8.584	0.510	1.353	−8.199	0.977	0.706	-	-	-	−8.405	0.690
UCP11E	-	-	-	-	-	-	−11.116	0.007	-	-	-

saDHFR = *S. aureus* dihydrofolate reductase (PDB ID: 2w9s); ecDHFR = *E. coli* dihydrofolate reductase (PDB ID: 7mym); caDHFR = *C. albicans* dihydrofolate reductase (PDB ID: 4hoe); hDHFR = human dihydrofolate reductase (PDB ID: 2w3a); ΔG = predicted binding energy; Kd = predicted dissociation constant; SI = selectivity index against human DHFR; UCP11E = 5-[3-(2,5-dimethoxy-4-phenylphenyl)but-1-yn-1-yl]-6 methylpyrimidine-2,4-diamine (reference inhibitor for caDHFR).

**Table 2:** Calculated physicochemical and drug-likeness properties of the investigated compounds.

Parameter	Ankaflavin	Monascin	Monascorubramine	Monascorubrin	Rubropunctamine	Rubropunctatin
Molecular weight (g/mol)	384.47	356.41	381.46	382.45	353.41	354.40
Rotatable bonds	8	6	8	8	6	6
H-bond acceptors	5	5	5	5	5	5
H-bond donors	0	0	0	0	0	0
Molar refractivity	107.25	97.63	109.59	106.77	99.19	97.16
TPSA (Å^2^)	69.67	69.67	73.33	69.67	73.33	69.67
WlogP	4.35	3.57	4.34	4.41	3.51	3.63
ESOL Log S	−4.56	−3.83	−4.68	−4.39	−3.97	−3.67
ESOL Class	Moderately soluble	Soluble	Moderately soluble	Moderately soluble	Soluble	Soluble
Lipinski violations	0	0	0	0	0	0
Bioavailability score	0.85	0.85	0.55	0.85	0.55	0.85

TPSA = topological polar surface area (apparent polarity); ESOL = Estimated Solubility. WlogP = octanol/water partition coefficient.

**Table 3:** Predicted pharmacokinetic parameters for the investigated natural compounds.

Parameter	Ankaflavin	Monascin	Monascorubramine	Monascorubrin	Rubropunctamine	Rubropunctatin
GI absorption	High	High	High	High	High	High
BBB permeant	Yes	Yes	Yes	Yes	Yes	Yes
Pgp substrate	No	No	No	No	No	No
CYP1A2 inhibitor	No	No	Yes	Yes	Yes	Yes
CYP2C19 inhibitor	Yes	Yes	Yes	Yes	Yes	Yes
CYP2C9 inhibitor	Yes	Yes	Yes	Yes	Yes	Yes
CYP2D6 inhibitor	Yes	No	Yes	Yes	No	No
CYP3A4 inhibitor	No	No	Yes	No	No	No
log Kp (cm/s)	−5.42	−6.02	−5.42	−5.58	−6.01	−6.18

Log Kp—skin permeation calculated using a QSPR method; Cytochrome P450 = CYP.

**Table 4:** Predicted toxicity parameters for the investigated natural compounds.

	Ankaflavin		Monascin		Monascorubramine		Monascorubrin		Rubropunctamine		Rubropunctatin	
Parameter	Class	*p*	Class	*p*	Class	*p*	Class	*p*	Class	*p*	Class	*p*
Hepatotoxicity	I	0.84	I	0.84	I	0.75	I	0.84	I	0.75	I	0.84
Neurotoxicity	I	0.76	I	0.76	I	0.78	I	0.76	I	0.78	I	0.76
Nephrotoxicity	I	0.54	I	0.54	A	0.51	I	0.54	A	0.51	I	0.54
Respiratory toxicity	A	0.80	A	0.80	A	0.79	A	0.80	A	0.79	A	0.80
Cardiotoxicity	A	0.51	A	0.51	I	0.59	A	0.51	I	0.59	A	0.51
Carcinogenicity	A	0.51	A	0.51	I	0.50	A	0.51	I	0.50	A	0.51
Immunotoxicity	A	0.97	A	0.94	A	0.99	I	0.94	A	0.84	I	0.97
Mutagenicity	I	0.80	I	0.80	I	0.78	I	0.80	I	0.78	I	0.80
Cytotoxicity	I	0.63	I	0.63	I	0.72	I	0.63	I	0.72	I	0.63
BBB toxicity	A	0.94	A	0.94	A	0.89	A	0.94	A	0.89	A	0.94
Ecotoxicity	I	0.55	I	0.55	I	0.51	I	0.55	I	0.51	I	0.55
LD_50_ (mg/kg)	250		250		416		130		2000		130	
Toxicity class	3		3		4		3		4		3	

A—active; I—inactive; LD_50_—median lethal dose; toxicity class 3—toxic if swallowed; toxicity class 4—harmful if swallowed; *p* = Probability.

**Table 5:** ESI-MS analysis of bioproducts obtained from *Monascus* sp.

ESI-MS Analysis of Alcoholic Extract from *Monascus purpureus*				
Peak Number	m/z	Abund %	Species/Compound	References
1	2	3	4	5
4	171.7083	5.22	Rubropunctatin	[30]
			Monaphilone B	
8	181.0159	9.41	Monascodilone	
14	199.0884	6.09	Monascusic acid F; Monacolin J; Monacolin M; Monacolin X hydroxy acid; Dehydromonacolin K hydroxy acid; Monacolin analogue; Monacolin L hydroxy acid; Monacolin X; Monascusic acid B; Monascusic acid A; Dehydromonacolin L	
16	215.6276	7	Monacolin J hydroxy acid	[30]
			Ankaflavin	
			Monasfluore B	
18	227.312	6.04	Daidzein; Dehydromonacolin L hydroxy acid; Dihydrocompactin; Dihydromonacolin L	[30]
21	242.378	5.89	Glycitein	[30]
22	245.5612	7.95	Monacolin L; Dihydrocompactin	[30]
24	255.0143	5.96	Daidzin; Daidzein	[30]
27	261.8811	5.22	Monascin	[30]
			Rubropunctatin	
28	265.0347	5.02	Monacolin analogue	[30]
29	265.1284	6.92	Monacolin analogue	[30]
33	295.1662	6.98	Monapurfluore A Monapurfluore B	[30]
36	305.7171	5.62	Dihydromonacolin K hydroxy acid Dihydromonacolin K	[30]
40	319.889	5.76	Hydroxy-monacolin K hydroxy acid Hydroxy-monacolin K hydroxy acid isomer	[30]
			Rubropunctamine	
44	327.8556	6.63	Monascorubrin	[30]
61	380.2135	6.89	Probably Monascorubramine	[29,30]
ESI-MS analysis of alcoholic extract from *Monascus ruber*				
Peak Number	m/z	Abund %	Species/Compound	References
1	170.4554	100	Tirozine; (±) Acid monascumic	[30]
3	181.0092	7.8	Monascodilone	[30]
4	184.6152	5.78	Citidine	[30]
5	184.6738	7.4	Citidine	[30]
6	187.3668	6.04	Citrinine	[30]
			New red pigment	
7	187.4972	10.79	Citrinine	[30]
			New red pigment	
12	201.9213	5.37	3α-hydroxy-3,5-dihyo-ML-236C; Dihydromonacolin L hydroxy acid; Monaphilone B; Monascusic acid B; Heptaketide; Dihydrocompactin;	[30]
16	211.0116	5.5	ML-236A; ML-236C	[30]
17	211.1204	7.34	ML-236A; ML-236C	[30]
18	216.7364	6.8	New red pigment	[30]
19	225.372	6.31	Monacolin J hydroxy acid; Monacolin J Monacolin M; Monacolin X hydroxy acid Dehydromonacolin K hydroxy acid Monacolin analogue; Monacolin X Dehydromonacolin L; Dehydromonacolin K	[30]
22	237.1678	6.54	Daidzein	[30]
26	249.4918	5.25	Ethyl ester of monacolin K Dehydromonacolin K	[30]
27	255.5845	6.46	Daidzin; Daidzein	[30]
29	261.8997	5.64	Monascin	[30]
			Rubropunctatin	
30	265.0534	5.02	Monacolin analogue	[30]
31	271.1478	6.91	Genistin; Genistein; Compactin acid	[30]
			New yellow pigment	
32	271.3223	7.54	Genistin; Genistein; Compactin acid	[30]
			New yellow pigment	
34	277.8281	5.17	Monapurfluore A; Monapurfluore B	[30]
35	277.9049	7.47	Monapurfluore A; Monapurfluore B	[30]
36	287.6625	6.24	3α-hydroxy-3,5-dihydromonacolin L; Dihydromonacolin J hydroxy acid; Dehydromonacolin L hydroxy acid; Monacolin L; Dihydromonacolin K hydroxy acid Dihydromonacolin K; Monaphilone A α,β-dehydrodihydromonacolin K	[30]
41	305.0892	5.55	Dihydromonacolin K hydroxy acid; Dihydromonacolin K	[30]
44	312.1581	9.4	New red pigment	[30]
45	315.7683	5.31	New yellow pigment	[30]
			Monaphilone A	
46	319.22	7.37	Hydroxy-monacolin K hydroxy acid Hydroxy-monacolin K hydroxy acid isomer	[30]
			Rubropunctamine	
51	337.0342	5.98	Monapurfluore A	[30]
			Rubropunctamine	
53	347.6858	5.52	Red pigment	[30]
54	347.7932	8.95	Red pigment	[30]
61	381.4657	7.77	Monascorubramine	[29,30]
81	475.0018	7.24	Unknown	[30]
ESI-MS analysis of total alcoholic extract from highly productive *Monascus ruber*				
Peak Number	m/z	Abund %	Compounds	References
4	181.007	8.53	Monascodilonely	[30]
6	184.0531	6.83	Citidina	
9	198.0307	5.17	(±) Acid monascumic Monascodilone	
10	201.1082	5.88	Ankaflavin	[30]
			Monascin	
			3α-hydroxy-3,5-dihyo-ML-236C Dihydromonacolin L hydroxy acid	
12	245.3446	5.53	Monacolin L; Dihydrocompactin	[30]
15	261.6577	6.35	Rubropunctatin	[30]
			Monascin	
20	295.9722	5.21	Monapurfluore A; Monapurfluore B	[30]
22	313.3179	5.37	Monascin	[30]
			Ethyl ester of monacolin K; Monasfluore B	
25	331.4616	5.59	Monascin	[30]
			Red pigment	[30]
28	354.2698	5.65	Rubropunctamine	[17]
29	358.1362	6.04	Ankaflavine	[30]
33	382.1032	8.28	Monascorubramine	[29,30]

**Table 6:** Extracts used in antimicrobial studies performed in vitro.

Sample	Extract Type	*Monascus* Strain	Crude Extract Concentration, mg/L
MR-red	Crude extract with red polyketides	*M. ruber*	110.208
MM-red		High productive *M. ruber*	1075
MP-red		*M. purpureus*	425.10
MR-orange	Crude extract with orange polyketides	*M. ruber*	400
MM-orange		High productive *M. ruber*	4800
MP-orange		*M. purpureus*	1600
MR-yellow	Crude extract with yellow polyketides	*M. ruber*	32.59
MM-yellow		High productive *M. ruber*	695.25
MP-yellow		*M. purpureus*	125.53

## Data Availability

The raw data supporting the conclusions of this article will be made available by the authors on request.

## References

[1] Jiao J., Liu Z., Zheng Y., Liu J. (2021). A novel application of *Monascus purpureus* in semi-soft cheese making. J. Food Process. Preserv..

[2] Yang Y., Xia Y., Li C., Wang G., Xiong Z., Song X., Zhang H., Wang M., Ai L. (2024). Metabolites, flavor profiles and ripening characteristics of *Monascus*-ripened cheese enhanced by *Lactobacillus salivarius* AR809 as adjunct culture. Food Chem..

[3] Zhang S., Wang T., Zhang Y., Song B., Pang X., Lv J. (2022). Effects of *Monascus* on Proteolysis, Lipolysis, and Volatile Compounds of Camembert-Type Cheese during Ripening. Foods.

[4] Lorrungruang C., Sinma K., Pantagrud P., Wannasirisuk S., Mahabandha K., Khucharoenphaisan K. (2014). Red Cheese Production from Soymilk by *Monascus purpureus* and *Lactobacillus casei*. J. Appl. Sci..

[5] Yuan X., Chen X., Virk M.S., Ma Y., Chen F. (2021). Effects of Various Rice-Based Raw Materials on Enhancement of Volatile Aromatic Compounds in *Monascus* Vinegar. Molecules.

[6] Takeshita R., Saigusa N., Teramoto Y. (2016). Production and antioxidant activity of alcoholic beverages made from various cereal grains using *Monascus purpureus* NBRC 5965. J. Inst. Brew..

[7] Klimek M., Wang S., Ogunkanmi A. (2009). Safety and efficacy of red yeast rice (*Monascus purpureus*) as an alternative therapy for hyperlipidemia. Pharm. Ther..

[8] Chen G., Chen W., Xu J., Ma G., Hu X., Chen G. (2023). The current trend and challenges of developing red yeast rice-based food supplements for hypercholesterolemia. J. Future Foods.

[9] Vitiello A., Izzo L., Castaldo L., d’Angelo I., Ungaro F., Miro A., Ritieni A., Quaglia F. (2023). The Questionable Quality Profile of Food Supplements: The Case of Red Yeast Rice Marketed Products. Foods.

[10] Fukami H., Higa Y., Hisano T., Asano K., Hirata T., Nishibe S. (2021). A Review of Red Yeast Rice, a Traditional Fermented Food in Japan and East Asia: Its Characteristic Ingredients and Application in the Maintenance and Improvement of Health in Lipid Metabolism and the Circulatory System. Molecules.

[11] Zhu B., Qi F., Wu J., Yin G., Hua J., Zhang Q., Qin L. (2019). Red Yeast Rice: A Systematic Review of the Traditional Uses, Chemistry, Pharmacology, and Quality Control of an Important Chinese Folk Medicine. Front. Pharmacol..

[12] Farkouh A., Baumgärtel C. (2019). Mini-review: Medication safety of red yeast rice products. Int. J. Gen. Med..

[13] Chaudhary V., Katyal P., Panwar H., Puniya A.K., Poonia A.K. (2022). Evaluating anti-microbial and anti-oxidative potential of red biopigment from *Monascus purpureus*. Environ. Conserv. J..

[14] Suraiya S., Jang W.J., Cho H.J., Bin Choi Y., Park H.D., Kim J.-M., Kong I.-S. (2019). Immunomodulatory Effects of *Monascus* spp.-Fermented Sacccharina japonica Extracts on the Cytokine Gene Expression of THP-1 Cells. Appl. Biochem. Biotechnol..

[15] Lin C.S., Hur H.F., Lin C.C. (2019). Antioxidant properties and antibacterial activity of fermented *Monascus purpureus* extracts. MOJ Food Process. Technol..

[16] Feng L.H., Li Y.Q., Sun G.J., Zhao X.Z. (2019). Antibacterial effect of orange *Monascus* pigment against *Staphylococcus aureus*. Acta Aliment..

[17] Kaur M., Goel M., Mishra R.C., Lahane V., Yadav A.K., Barrow C.J. (2023). Characterization of the Red Biochromes Produced by the Endophytic Fungus *Monascus purpureus* CPEF02 with Antimicrobial and Antioxidant Activities. Fermentation.

[18] Zhao G., Li Y., Yang J., Cui K. (2016). Antibacterial characteristics of orange pigment extracted from *Monascus* pigments against *Escherichia coli*. Czech J. Food Sci..

[19] Vendruscolo F., Tosin I., Giachini A.J., Schmidell W., Ninow J.L. (2014). Antimicrobial Activity of *Monascus* Pigments. J. Food Process. Preserv..

[20] Gajalakshmi P., Raja A., Banu H.M.V. (2019). Efficacy of polyketide pigment produced by *Monascus purpureus* and its biological activity. Nutrafoods.

[21] Bouksir K., Kazzaz M., Fehri H.F., Bouziane H., Bouksir H., El Haskouri F. (2018). *Monascus* ruber: A new of onychomycosis in the north of Morocco (Tetouan). J. Mycol. Médicale.

[22] Tu C.-T., Chen Y.-P., Yu M.-C., Hwang I.-R., Wu D.Y., Liaw L.-L. (2016). Characterization and expression of the antifungal protein from *Monascus pilosus* and its distribution among various *Monascus* species. J. Biosci. Bioeng..

[23] Chaudhary V., Katyal P., Poonia A.K., Kaur J., Puniya A.K., Panwar H. (2022). Natural pigment from *Monascus*: The production and therapeutic significance. J. Appl. Microbiol..

[24] Moussa L.A., Azeiz A.Z. (2017). Effect of media composition on citrinin and bio-pigments production by *Monascus ruber*. J. Appl. Biol. Biotechnol..

[25] Egea M.B., Dantas L.A., Sousa T.L.D., Lima A.G., Lemes A.C. (2023). The potential, strategies, and challenges of *Monascus* pigment for food application. Front. Sustain. Food Syst..

[26] Carvalho J.C., Oishi B.O., Woiciechowski A.L., Pandey A., Babitha S., Soccol C.R. (2007). Effect of substrates on the production of *Monascus* biopigments by solid-state fermentation and pigment extraction using different solvents. Indian J. Biotechnol..

[27] Huang T., Tan H., Chen G., Wang L., Wu Z. (2017). Rising temperature stimulates the biosynthesis of water-soluble fluorescent yellow pigments and gene expression in *Monascus ruber* CGMCC10910. AMB Express.

[28] Chen S., Su D.X., Gao M.X., Zhang J.-L., Liu Y.-B., Wu Q.-H., Yang H.-L., Li L. (2021). A facile macroporous resin-based method for separation of yellow and orange *Monascus* pigments. Food Sci. Biotechnol..

[29] Yuliana A., Rahmawati L., Adlina S. (2021). Isolation and Identification of New Pigment from *Monascus purpureus*. Ad-Dawaa’ J. Pharm. Sci..

[30] Liang J.-X., Zhang Q.-Q., Huang Y.-F., Pang H.Q., Liu X.-G., Gao W., Li P., Yang H. (2019). Comprehensive chemical profiling of *Monascus*-fermented rice product and screening of lipid-lowering compounds other than monacolins. J. Ethnopharmacol..

[31] Adin S.N., Gupta I., Panda B.P., Mujeeb M. (2023). Monascin and ankaflavin-Biosynthesis from *Monascus purpureus*, production methods, pharmacological properties: A review. Biotechnol. Appl. Biochem..

[32] Srianta I., Zubaidah E., Estiasih T., Yamada M.H. (2016). Comparison of *Monascus purpureus* growth, pigment production and composition on different cereal substrates with solid-state fermentation. Biocatal. Agric. Biotechnol..

[33] Koli S.H., Suryawanshi R.K., Mohite B.V., Patil S.V. (2019). Prospective of *Monascus* Pigments as an Additive to Commercial Sunscreens. Nat. Prod. Commun..

[34] Majhi S., Dhale M.A., Honganoor Puttananjaiah M. (2023). Inhibitory effect of *Monascus purpureus* pigment extracts against fungi and mechanism of action. Front. Sustain. Food Syst..

[35] Yuliana A., Hilman-Fitriaji S.P., Mukhaufillah K.S., Rizkuloh L.R. (2020). In Silico Study on Testing Antidiabetic Compounds Candidate from Azaphilone *Monascus* sp.. Microbiol. Indones..

[36] Singgih M., Permana B., Maulidya S.A., Yuliana A. (2019). Studi In Silico Metabolit Sekunder Kapang *Monascus* sp. sebagai Kandidat Obat Antikolesterol dan Antikanker. ALCHEMY J. Penelit. Kim..

[37] Rizkuloh L.R., Pratita A.T., Mediana M., Yuliana A. (2021). In silico study in toxicity parameters of Pigment Derivated Compounds of *Monascus* sp. mold as a cervical anti-cancer drugs candidate. J. Teknol. Lab..

[38] Zain D.N., Yuliana A. (2023). In Silico Study of *Monascus* sp. Pigment Derivatives as Anticardiovascular Candidate. J. Ilm. Farm..

[39] Wu L., Zhou K., Yang Z., Li J., Chen G., Wu Q., Lv X., Hu W., Rao P., Ai L. (2022). Monascuspiloin from *Monascus*-Fermented Red Mold Rice Alleviates Alcoholic Liver Injury and Modulates Intestinal Microbiota. Foods.

[40] Hashimoto T., Ozaki A., Hakariya H., Takahashi K., Tanimoto T. (2024). The *Beni-Koji* scandal and Japan’s unique health food system. Lancet.

[41] Luță E.A., Biță A., Moroșan A., Mihaiescu D.E., Ghica M., Mihai D.P., Olaru O.T., Deculescu-Ioniță T., Duțu L.E., Popescu M.L. (2022). The Influence of Phytosociological Cultivation and Fertilization on Polyphenolic Content of *Menthae* and *Melissae folium* and Evaluation of Antioxidant Properties through In Vitro and In Silico Methods. Plants.

[42] Ungureanu A.R., Chițescu C.L., Luță E.A., Moroșan A., Mihaiescu D.E., Mihai D.P., Costea L., Ozon E.A., Fița A.C., Balaci T.D. (2023). Outlook on Chronic Venous Disease Treatment: Phytochemical Screening, In Vitro Antioxidant Activity and In Silico Studies for Three Vegetal Extracts. Molecules.

[43] Heaslet H., Harris M., Fahnoe K., Sarver R., Putz H., Chang J., Subramanyam C., Barreiro G., Miller J.R. (2009). Structural comparison of chromosomal and exogenous dihydrofolate reductase from *Staphylococcus aureus* in complex with the potent inhibitor trimethoprim. Proteins.

[44] Krucinska J., Lombardo M.N., Erlandsen H., Estrada A., Si D., Viswanathan K., Wright D.L. (2022). Structure-guided functional studies of plasmid-encoded dihydrofolate reductases reveal a common mechanism of trimethoprim resistance in Gram-negative pathogens. Commun. Biol..

[45] Daina A., Michielin O., Zoete V. (2017). SwissADME: A free web tool to evaluate pharmacokinetics, drug-likeness and medicinal chemistry friendliness of small molecules. Sci. Rep..

[46] Banerjee P., Kemmler E., Dunkel M., Preissner R. (2024). ProTox 3.0: A webserver for the prediction of toxicity of chemicals. Nucleic Acids Res..

[47] Burloiu A.M., Manda G., Lupuliasa D., Socoteanu R.P., Mihai D.P., Neagoe I.V., Anghelache L.-I., Surcel M., Anastasescu M., Olariu L. (2024). Assessment of Some Unsymmetrical Porphyrins as Promising Molecules for Photodynamic Therapy of Cutaneous Disorders. Pharmaceuticals.

[48] Baker D.J., Beddell C.J., Champness J.N., Goodford P.J., Norrington F.E.A., Smith D.R., Stammers D.K. (1981). The binding of trimethoprim to bacterial dihydrofolate reductase. FEBS Lett..

[49] Ghany L.M.A.A., Ryad N., Abdel-Aziz M.S., El-Lateef H.M.A., Zaki I., Beshay B.Y. (2024). Design, synthesis, antimicrobial evaluation, and molecular modeling of new sulfamethoxazole and trimethoprim analogs as potential DHPS/DHFR inhibitor. J. Mol. Struct..

[50] Wróbel A., Arciszewska K., Maliszewski D., Drozdowska D. (2020). Trimethoprim and other nonclassical antifolates an excellent template for searching modifications of dihydrofolate reductase enzyme inhibitors. J. Antibiot..

[51] Gleckman R., Blagg N., Joubert D.W. (1981). Trimethoprim: Mechanisms of action, antimicrobial activity, bacterial resistance, pharmacokinetics, adverse reactions, and therapeutic indications. Pharmacother. J. Hum. Pharmacol. Drug Ther..

[52] World Health Organization World Health Organization Model List of Essential Medicines: 22nd List (2021). https://iris.who.int/handle/10665/345533.

[53] Agboyibor C., Kong W.B., Chen D., Zhang A.-M., Niu S.-Q. (2018). *Monascus* pigments production, composition, bioactivity and its application: A review. Biocatal. Agric. Biotechnol..

[54] Zaharie M.-G.O., Radu N., Pirvu L., Bostan M., Voicescu M., Begea M., Constantin M., Voaides C., Babeanu N., Roman V. (2022). Studies Regarding the Pharmaceutical Potential of Derivative Products from Plantain. Plants.

[55] Babeanu N., Radu N., Enascuta C.-E., Alexandrescu E., Ganciarov M., Mohammed M.S.O., Suica-Bunghez I.R., Senin R., Ursu M., Bostan M. (2022). Obtaining and Characterizing Composite Biomaterials of Animal Resources with Potential Applications in Regenerative Medicine. Polymers.

